# Cutaneous Angiosarcoma Postmastectomy (Stewart-Treves Syndrome)

**DOI:** 10.5334/jbsr.1624

**Published:** 2018-10-01

**Authors:** Bert Degrieck, Ilse Crevits

**Affiliations:** 1 UZ Gent, BE; 2 AZ Delta Roeselare, BE

**Keywords:** cutaneous angiosarcoma, Stewart-Treves Syndrome, MRI

## Case report

A 70-year-old woman underwent a right-sided mastectomy and axillary lymph node excision because of invasive adenocarcinoma in 2007. She received additional radiotherapy on the mastectomy site and the right axilla. Extensive lymphedema of the right arm developed and formed ecchymosis, which persisted despite a microsurgical lymphovenous derivation in 2009. On clinical examination in 2017, a painful nodular and purpuric transformation of the skin was noted. Because of increasing pain in the upper arm, a biopsy of the skin was performed, revealing a cutaneous epitheloid angiosarcoma. The patient was referred for magnetic resonance imaging (MRI) of the right upper arm to evaluate local extent.

**Figure 1:**
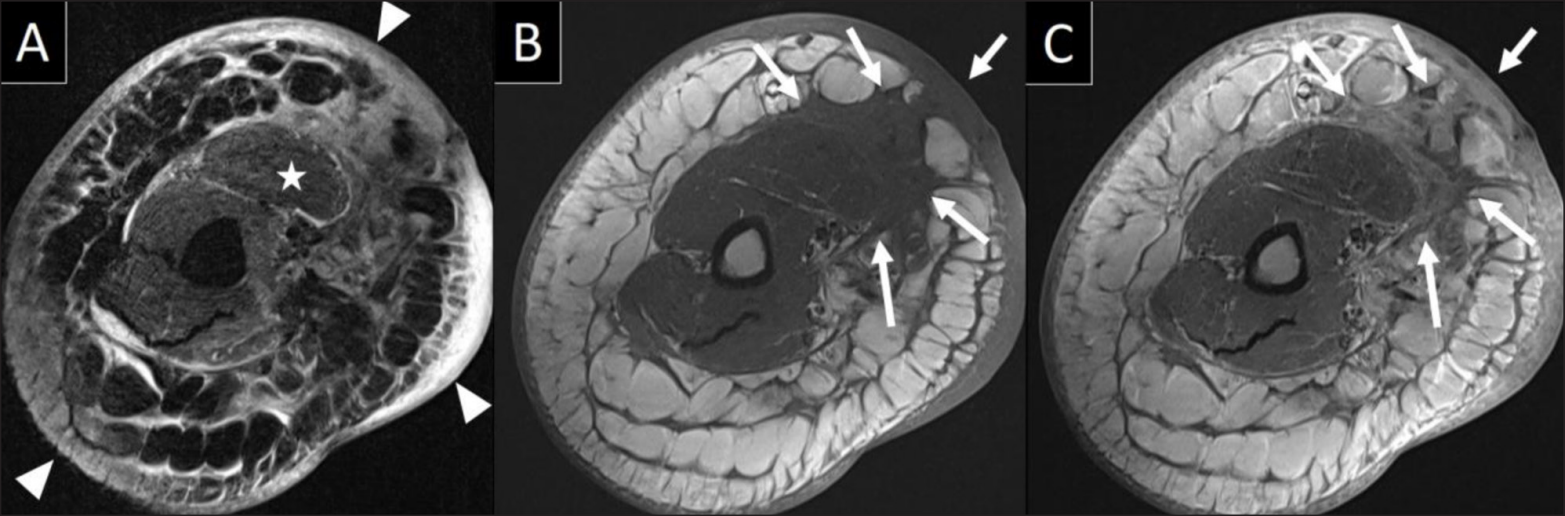
MRI of the right upper arm: axial TIR **(a)**, T1 before **(b)** and T1 after **(c)** intravenous contrast. Diffuse thickening of the cutis and subcutis (arrowheads in 1a) as well as muscle edema (asterisk in 1a) can be seen. There is an amorphous heterogeneous enhancing mass (arrows in 1b and 1c) extending from the superficial cutis to the biceps muscle, with spicular infiltration reaching the neurovascular bundle.

Axial Tau Inversion Recovery (TIR) and T1-weighted images of the right upper arm (Figure 1a and 1b) show a diffusely thickened cutis and subcutis with extensive lymphedema (arrowheads), as well as muscle edema (asterisk). There is an amorphous mass extending from the ventral cutis to the biceps muscle (arrows in Figure 1b and 1c) with spicular infiltration reaching the neurovascular bundle. This mass has an intermediate signal on T1-weighted and a heterogeneous high signal on TIR images, and shows heterogeneous contrast enhancement (Figure 1c).

The patient was treated with an amputation of the affected arm. Follow-up CT examination revealed progressive disease with new subcutaneous and intramuscular metastases in the right hemithorax, and the additional follow up MRI revealed diffuse skeletal metastasis.

## Discussion

Stewart Treves syndrome or angiosarcoma secondary to chronic lymphatic obstruction is a very rare and lethal disease. It is most commonly seen in the upper arm of female breast cancer patients who underwent mastectomy and axillary lymph node excision. [1] Besides chronic lymphedema, additional factors such as radiation therapy may increase the risk. The incidence of Stewart Treves syndrome has significantly decreased with the improvement of operative and radiation therapy techniques and the use of chemotherapy.

Typically, these lesions have intermediate signal intensity on T1-weighted images and high signal on images obtained with long repetition times on MRI. They show aggressive infiltration of the surrounding tissues. They may also show serpentine vascular channels, most often in the lesion periphery, a feature rarely seen in other soft tissue sarcomas. These lesions have no fat overgrowth.

Imaging features on CT are nonspecific, showing a soft tissue mass with attenuation similar to muscle that is typically markedly enhancing.

US descriptions are rare, showing complex hypo- or hyperechoic lesions with cystic components (resulting from haemorrhage). Doppler US often shows arteriovenous shunting.

The treatment of choice is surgical resection of the affected limb, though local recurrent and distant metastasis are frequent due to their aggressive nature, and despite radical treatment, prognosis is poor.

## References

[1] Sharma, A and Schwartz, RA. Stewart-Treves syndrome: Pathogenesis and management. J Am Acad Dermatol. 2012 12; 67(6): 1342–8. DOI: 10.1016/j.jaad.2012.04.028 22682884

